# Racial/Ethnic and Socioeconomic Disparities in Management of Incident Paroxysmal Atrial Fibrillation

**DOI:** 10.1001/jamanetworkopen.2021.0247

**Published:** 2021-02-26

**Authors:** Lauren A. Eberly, Lohit Garg, Lin Yang, Timothy M. Markman, Ashwin S. Nathan, Nwamaka D. Eneanya, Sanjay Dixit, Francis E. Marchlinski, Peter W. Groeneveld, David S. Frankel

**Affiliations:** 1 Cardiovascular Division, Perelman School of Medicine at the University of Pennsylvania, Philadelphia; 2 Center for Cardiovascular Outcomes, Quality, and Evaluative Research, University of Pennsylvania, Philadelphia; 3 Penn Cardiovascular Center for Health Equity and Social Justice, University of Pennsylvania, Philadelphia; 4 Renal-Electrolyte and Hypertension Division, Perelman School of Medicine at the University of Pennsylvania, Philadelphia; 5 Center for Clinical Epidemiology and Biostatistics, Perelman School of Medicine at the University of Pennsylvania, Philadelphia; 6 Corporal Michael J. Crescenz Veterans Affairs Medical Center, Philadelphia, Pennsylvania; 7 Division of General Internal Medicine, Perelman School of Medicine at the University of Pennsylvania, Philadelphia

## Abstract

**Importance:**

In patients with paroxysmal atrial fibrillation (AF), rhythm control with either antiarrhythmic drugs (AADs) or catheter ablation has been associated with decreased symptoms, prevention of adverse remodeling, and improved cardiovascular outcomes. Adoption of advanced cardiovascular therapeutics, however, is often slower among patients from racial/ethnic minority groups and those with lower income.

**Objective:**

To ascertain the cumulative rates of AAD and catheter ablation use for the management of paroxysmal AF and to investigate for the presence of inequities in AF management by evaluating the association of race/ethnicity and socioeconomic status with their use in the United States.

**Design, Setting, and Participants:**

This cohort study obtained inpatient, outpatient, and pharmacy claims data from the Optum Clinformatics Data Mart between October 1, 2015, and June 30, 2019. Adult patients (aged ≥18 years) in the database with a diagnosis of incident paroxysmal AF were identified. Patients were excluded if they did not have continuous insurance enrollment for at least 1 year before and at least 6 months after study entry.

**Exposures:**

Race/ethnicity and zip code–linked median household income.

**Main Outcomes and Measures:**

Treatment with a rhythm control strategy, and catheter ablation specifically, among those who received rhythm control. Multivariable logistic regression models were used to assess the association of race/ethnicity and zip code–linked median household income with a rhythm control strategy (AADs or catheter ablation) vs a rate control strategy as well as with catheter ablation vs AADs among those receiving rhythm control.

**Results:**

Of the 109 221 patients who met the inclusion criteria, 55 185 were men (50.5%) and 73 523 were White (67.3%), with a median (interquartile range) age of 75 (68-82) years. A total of 86 359 patients (79.1%) were treated with rate control, 19 362 patients (17.7%) with AADs, and 3500 (3.2%) with catheter ablation. Between 2016 and 2019, the cumulative percentage of patients treated with catheter ablation increased from 1.6% to 3.8%. In multivariable analyses, Black race (adjusted odds ratio [aOR], 0.89; 95% CI, 0.83-0.94; *P* < .001) and lower zip code–linked median household income (aOR for <$50 000: 0.83 [95% CI, 0.79-0.87; *P* < .001]; aOR for $50 000-$99 999: 0.92 [95% CI, 0.88-0.96; *P* = <.001] compared with ≥$100 000) were independently associated with lower use of rhythm control. Latinx ethnicity (aOR, 0.73; 95% CI, 0.60-0.89; *P* = .002) and lower zip code–linked median household income (aOR for <$50 000: 0.61 [95% CI, 0.54-0.69; *P* < .001]; aOR for $50 000-$99 999: 0.81 [95% CI, 0.72-0.90; *P* < .001] compared with ≥$100 000) were independently associated with lower catheter ablation use among those receiving rhythm control.

**Conclusions and Relevance:**

This study found that despite increased use of rhythm control strategies for treatment of paroxysmal AF, catheter ablation use remained low and patients from racial/ethnic minority groups and those with lower income were less likely to receive rhythm control treatment, especially catheter ablation. These findings highlight inequities in paroxysmal AF management based on race/ethnicity and socioeconomic status.

## Key Points

**Question:**

Are race/ethnicity and socioeconomic status associated with use of a rhythm control strategy (antiarrhythmic drugs and catheter ablation), and specifically catheter ablation, among patients with paroxysmal atrial fibrillation (AF) in the United States?

**Findings:**

In this cohort study of 109 221 patients with incident paroxysmal AF, Black race and lower zip code–linked median household income were associated with less use of a rhythm control strategy, and Latinx ethnicity and lower zip code–linked median household income were associated with less use of catheter ablation.

**Meaning:**

Results of this study suggest that racial/ethnic and socioeconomic inequities may be present in management of paroxysmal AF in the United States.

## Teaser

This cohort study examines inpatient, outpatient, and pharmacy claims to identify use of antiarrhythmic drugs and catheter ablation among commercially insured adults with paroxysmal atrial fibrillation.

## Introduction

Atrial fibrillation (AF), the most common sustained cardiac arrhythmia, is associated with substantial morbidity and mortality, including frequent hospitalizations, heart failure, and thromboembolic events.^1^ Although a rate control strategy is a reasonable approach for managing AF in many patients, certain factors clearly favor a rhythm control strategy.^2^ For patients with symptomatic paroxysmal AF, a rhythm control strategy has been shown to improve quality of life.^3^ Paroxysmal AF often progresses to persistent AF, and a rhythm control strategy may halt the accompanying structural and electrical adverse remodeling.^2,4^ Furthermore, recent evidence suggests that early pursuit of a rhythm control strategy is associated with improved cardiovascular outcomes.^5^ Maintenance of sinus rhythm may be achieved with the use of antiarrhythmic drugs (AADs) or catheter ablation.^2,4,6^ Current guidelines recommend catheter ablation as a first-line treatment for symptomatic paroxysmal AF.^2^ Among patients with heart failure with reduced ejection fraction (HFrEF) and AF, catheter ablation has been shown to decrease mortality and heart failure hospitalizations as well as increase left ventricular ejection fraction.^7,8,9,10^

Inequitable care delivery based on race/ethnicity is pervasive, and decreased access to cardiovascular therapies among patients from racial/ethnic minority groups and those with lower income has been demonstrated.^11^ There is decreased adoption of advanced cardiovascular therapeutics among Black, Latinx, and low-income patients.^12,13,14,15^ Demographic studies have shown that patients with AF who underwent catheter ablation were mostly White and male.^16^ Racial and sex inequities in receipt of certain AF-related therapies were demonstrated among Medicare patients.^17^ However, broader, larger-scale studies are lacking that evaluate the association of race and socioeconomic status with use of a rhythm control strategy, particularly catheter ablation, among commercially insured patients with paroxysmal AF. The objectives of this cohort study were to ascertain the cumulative rates of AAD and catheter ablation use for the management of paroxysmal AF and to investigate the presence of inequities in AF management by evaluating the association of race/ethnicity and socioeconomic status with their use in the United States.

## Methods

The University of Pennsylvania Institutional Review Board determined that this research was exempt from the regulatory requirements of the federal Common Rule because no protected health information was used. We followed the Strengthening the Reporting of Observational Studies in Epidemiology (STROBE) reporting guideline.

### Study Data and Cohort

Data were obtained by contractual agreement from the Optum Clinformatics Data Mart, a deidentified database of administrative claims by members of a commercial insurance plan and Medicare Advantage. This database consists of inpatient, outpatient, and pharmacy claims from more than 15 million patients annually. Demographic variables at enrollment, such as age, sex, and race/ethnicity, are available for each member. Socioeconomic data, including median household income, are available through zip code–linked enrollment data from the US Census Bureau.

We identified adult patients (aged ≥18 years) in the database with a diagnosis of paroxysmal AF documented with *International Statistical Classification of Diseases and Related Health Problems, Tenth Revision (ICD-10)* code I48.0 between October 1, 2015, and June 30, 2019. We required the diagnosis to be coded on at least 2 occasions on separate dates either in the inpatient or outpatient setting. Patients entered the study cohort on the date of the second coded diagnosis for paroxysmal AF. Patients were excluded if they did not have continuous insurance enrollment for at least 1 year before and at least 6 months after study entry to ensure that comorbidities, clinical data, and prescription claims could be accurately identified. In addition, patients were excluded if they had no pharmacy claims for medication for 1 year before the study period to ensure that medication use was accurately captured in the data set.

To ensure that only incident paroxysmal AF was analyzed, we excluded patients with preexisting AF diagnoses based on *International Classification of Diseases, Ninth Revision (ICD-9)* code 427.31 before the *ICD-10* diagnosis as well as patients with previous prescription claims for amiodarone hydrochloride, disopyramide phosphate, dofetilide, dronedarone hydrochloride, flecainide acetate, mexiletine hydrochloride, propafenone hydrochloride, quinidine, or sotalol hydrochloride before the *ICD-10* diagnosis.

Rhythm control was defined by a prescription claim for 1 or more AAD after cohort entry (ie, amiodarone, disopyramide, dofetilide, dronedarone, flecainide, mexiletine, propafenone, quinidine, or sotalol) or by a *Current Procedural Terminology* (*CPT*) code for catheter ablation (93656). Rate control was defined as the absence of rhythm control. All patients who had a *CPT* code for catheter ablation, regardless of AAD use, were included in the catheter ablation group. The mean number of outpatient cardiology visits per 12 months after cohort entry was ascertained on the basis of having a visit with a cardiology practitioner with *CPT* codes 99201-99205 or 99211-99215.

### Statistical Analysis

Summary statistics for patient characteristics are presented as medians with interquartile ranges (IQRs) or means with SDs for continuous data and as a total number and percentages for categorical data. Continuous variables were compared using an unpaired, 2-tailed *t* test, and categorical variables were compared using the χ^2^ test. Cumulative yearly rates of AAD and catheter ablation use were calculated by dividing the total number of patients treated until each time point starting in 2016 (given that only 3 months were represented in 2015) by the total number of patients in the cohort at that time. We also assessed cumulative yearly rates of AAD and catheter ablation use in patients with a diagnosis of HFrEF (*ICD-10* codes I50.2, I50.21, I50.22, I50.23, I50.4, I50.41, I40.42, I50.43, I50.82, and I50.84).

To assess the association of race/ethnicity, sex, and socioeconomic status with the use of rhythm control strategy (both AADs and catheter ablation), we estimated multivariable logistic regression models with the use of AADs or catheter ablation as the dependent variable and with demographic factors (age, sex, race/ethnicity, region of residence, zip code–linked median household income, and health insurance subset), clinical factors (anticoagulation status, number of outpatient cardiology visits per 12 months), and comorbidities (number of Elixhauser comorbidities,^18^ coronary artery disease, chronic kidney disease, stroke or transient ischemic attack, obesity, hypertension, diabetes, peripheral vascular disease, dyslipidemia, HFrEF, and heart failure with preserved ejection fraction) as the independent variables.

To assess the association of race/ethnicity, sex, and socioeconomic status with the use of catheter ablation among those in whom a rhythm control strategy was selected, we included patients who had either a prescription for AADs or a *CPT* code for catheter ablation during the study period. We estimated multivariable logistic regression models with the use of catheter ablation as the dependent variable and the aforementioned independent variables. Candidate variables included in the model were selected a priori on the basis of pathophysiological plausibility or because of significance on univariate analysis. Estimated adjusted odds ratios (aORs) are reported with 95% CIs.

Among those who underwent catheter ablation, the number of AADs prescribed after paroxysmal AF diagnosis and before catheter ablation was compared between racial/ethnic, sex, and zip code–linked median household income groups. Statistical analyses were performed using SAS, version 9.4 (SAS Institute, Inc). All statistical testing was 2-tailed, with *P* < .05 designated as statistically significant.

## Results

**Figure 1.**
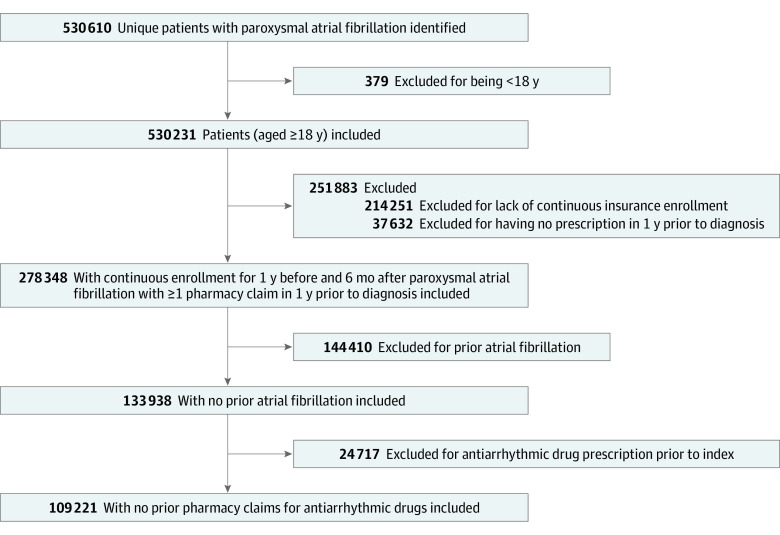
Selection of Study Population Antiarrhythmic drug includes amiodarone hydrochloride, disopyramide phosphate, dofetilide, dronedarone hydrochloride, flecainide acetate, mexiletine hydrochloride, propafenone hydrochloride, quinidine, and sotalol hydrochloride.

A total of 109 221 patients met the inclusion criteria (Figure 1), with 86 359 patients (79.1%) treated with a rate control strategy, and 19 362 (17.7%) treated with AADs, and 3500 (3.2%) treated with catheter ablation. The median (IQR) age of patients was 75 (68-82) years and 54 024 were female (49.5%) and 55 185 were male (50.5%) patients. In 12 patients, sex was missing or unknown. The cohort included 73 523 White (67.3%), 10 192 Black (9.3%), and 8053 Latinx (7.4%) patients. The zip code–linked median household income for 33 279 patients (30.5%) was less than $50 000 and for 19 530 patients (17.9%) was $100 000 or greater. Baseline demographic, socioeconomic, and clinical characteristics for all included patients with paroxysmal AF by treatment strategy are summarized in Table 1. Among those in the rate control group, 54 877 patients (63.5%) were treated with a β-blocker, 14 105 (16.3%) with a nondihydropyridine calcium channel blocker, and 3614 (4.2%) with digoxin.

**Table 1.** Baseline Demographic, Socioeconomic, and Clinical Characteristics for All Patients With Paroxysmal Atrial Fibrillation

Variable	No. (%)			*P* value
	Rate control group (n = 86 359)	AAD group (n = 19 362)	Catheter ablation group (n = 3500)	
Age, median (IQR), y	76 (69-83)	74 (68-80)	68 (60-73)	<.001
Sex^a^				
Male	43 217 (50.0)	9863 (50.9)	2105 (60.1)	<.001
Female	43 134 (49.9)	9495 (49.0)	1395 (39.9)	<.001
Race/ethnicity				<.001
Asian	1729 (2.0)	341 (1.8)	73 (2.1)	
Black	8136 (9.4)	1826 (9.4)	230 (6.6)	
Latinx^b^	6460 (7.5)	1428 (7.4)	165 (4.7)	
White	57 800 (66.9)	13 179 (68.1)	2544 (72.7)	
Unknown	12 234 (14.2)	2588 (13.4)	488 (13.9)	
Region				<.001
Midwest	18 873 (21.9)	4336 (22.4)	802 (22.9)	
Northeast	10 736 (12.4)	1574 (8.1)	327 (9.3)	
South	36 562 (42.3)	8982 (46.4)	1586 (45.3)	
West	19 953 (23.1)	4425 (22.9)	781 (22.3)	
Zip code–linked median household income, $				<.001
<50 000	26 878 (31.1)	5779 (29.8)	622 (17.8)	
50 000-99 999	25 363 (29.4)	5843 (30.2)	1060 (30.3)	
≥100 000	14 777 (17.1)	3620 (18.7)	1133 (32.4)	
Insurance type				<.001
Commercial	12 121 (14.0)	3689 (19.1)	1446 (41.3)	
Medicare Advantage	74 238 (86.0)	15 673 (80.9)	2054 (58.7)	
Comorbidity				
Coronary artery disease	42 976 (49.8)	10 530 (54.4)	1275 (36.4)	<.001
Stroke/TIA	16 132 (18.7)	3056 (15.8)	319 (9.1)	<.001
Chronic kidney disease	28 663 (33.2)	6342 (32.8)	531 (15.2)	<.001
Dyslipidemia	72 961 (84.5)	16 560 (85.5)	2757 (78.8)	<.001
Obesity	29 492 (34.2)	7312 (37.8)	1309 (37.4)	<.001
Hypertension	77 644 (89.9)	17 530 (90.5)	2776 (79.3)	<.001
Diabetes	37 030 (42.9)	8365 (43.2)	996 (28.5)	<.001
Peripheral vascular disease	32 095 (37.2)	7318 (37.8)	685 (19.6)	<.001
HFrEF	14 150 (16.4)	5336 (27.6)	557 (15.9)	<.001
HFpEF	14 891 (17.2)	4882 (25.2)	457 (13.1)	<.001
HF hospitalization^c^	8927 (10.3)	3738 (19.3)	348 (9.9)	<.001
No. of Elixhauser comorbidities				<.001
0-1	5075 (5.9)	979 (5.1)	534 (15.3)	
2-3	13 703 (15.9)	2771 (14.3)	959 (27.4)	
4-6	26 370 (30.5)	6138 (31.7)	1217 (34.8)	
≥7	41 211 (47.7)	9474 (48.9)	790 (22.6)	
CHA_2_DS_2_-VASc score category				<.001
Low risk: 0-1	4612 (5.3)	1068 (5.5)	726 (20.7)	
Medium risk: 2-5	54 693 (63.3)	12 700 (65.6)	2438 (69.7)	
High risk: >5	27 054 (31.3)	5594 (28.9)	336 (9.6)	
Anticoagulation use				
Warfarin sodium	12 740 (14.8)	3459 (17.9)	396 (11.3)	<.001
DOAC	34 056 (39.4)	11 263 (58.2)	2819 (80.5)	<.001
No anticoagulation	41495 (48.0)	5463 (28.2)	438 (43.4)	<.001
Medications				
β-Blocker	54 877 (63.5)	15 666 (80.9)	2625 (75.0)	<.001
Digoxin	3614 (4.2)	1009 (5.2)	171 (4.9)	<.001
Nondihydropyridine calcium channel blocker	14 105 (16.3)	3952 (20.4)	978 (27.9)	<.001
No. of cardiology visits per 12 mo				<.001
0	16 599 (19.2)	2056 (10.6)	128 (3.7)	
1	34 690 (40.2)	7244 (37.4)	986 (28.2)	
>1	35 070 (40.6)	10 062 (52.0)	2386 (68.2)	

Abbreviations: AAD, antiarrhythmic drug; CHA_2_DS_2_-VASc score, congestive heart failure, hypertension, age 75 years or older (2 points), diabetes, history of stroke, transient ischemic attack, or systemic thromboembolism (2 points), vascular disease, age 65 to 74 years, and female sex; DOAC, direct oral anticoagulant; HF, heart failure; HFpEF, heart failure with preserved ejection fraction; HFrEF, heart failure with reduced ejection fraction; IQR, interquartile range; TIA, transient ischemic attack.

^a^ In 12 patients, sex was missing or unknown (8 in the rate control group and 4 in the AAD group).

^b^ Latinx is a gender-neutral term describing persons of Latin American origin or descent.

^c^ Within the past 12 months.

**Figure 2.**
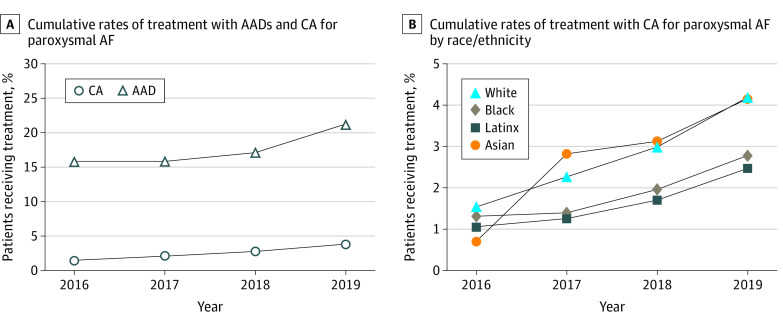
Cumulative Rates of Treatment AAD indicates antiarrhythmic drug; AF, atrial fibrillation; and CA, catheter ablation.

The cumulative rate of AAD use increased from 15.8% in 2016 to 21.3% in 2019, whereas the cumulative percentage of patients treated with catheter ablation increased from 1.6% in 2016 to 3.8% in 2019 (Figure 2A). The cumulative percentage of AAD use increased from 12.9% in 2016 to 19.4% in 2019 among Asian patients, from 16.6% to 22.1% among Black patients, from 16.9% to 21.4% among Latinx patients, and from 15.9% to 21.7% among White patients. Cumulative rates of catheter ablation use by race/ethnicity are depicted in Figure 2B. The cumulative percentage of catheter ablation use increased from 0.7% in 2016 to 4.2% in 2019 among Asian patients, from 1.3% to 2.8% among Black patients, from 1.1% to 2.5% among Latinx patients, and from 1.5% to 4.2% among White patients.

Among those with HFrEF (n = 20 043), 14 150 patients (70.6%) were treated with a rate control strategy, followed by 5336 patients (26.6%) who used AADs and 557 (2.8%) who received catheter ablation (*P* < .001). The cumulative rate of patients with HFrEF who were treated with AADs increased from 24.6% in 2016 to 34.3% in 2019, whereas the cumulative rate of catheter ablation use increased from 1.0% in 2016 to 3.6% in 2019 (eFigure in the Supplement). Cumulative rates of catheter ablation among patients with HFrEF by race/ethnicity are shown in the eFigure in the Supplement.

### Rhythm Control vs Rate Control

In multivariable analyses (Table 2), female sex was independently associated with higher use of a rhythm control strategy (aOR, 1.08; 95% CI, 1.04-1.12; *P* < .001). Lower zip code–linked median household income was associated with less use of rhythm control for those with an income of less than $50 000 (aOR, 0.83; 95% CI, 0.79-0.87; *P* < .001) and for those with an income of $50 000 to $99 999 (aOR, 0.92; 95% CI, 0.88-0.96; *P* = <.001) compared with an income of $100 000 or more. Black race was also independently associated with less rhythm control use (aOR, 0.89; 95% CI, 0.83-0.94; *P* < .001). Heart failure with preserved ejection fraction (aOR, 1.41; 95% CI, 1.35-1.48; *P* < .001) and HFrEF (aOR, 1.67; 95% CI, 1.59-1.75; *P* < .001) were both associated with higher rhythm control use. Having 1 cardiology visit per 12 months (aOR, 1.62; 95% CI, 1.52-1.73; *P* < .001) and more than 1 cardiology visit per 12 months (aOR, 2.36; 95% CI, 2.21-2.52; *P* < .001) were also independently associated with increased use of rhythm control.

**Table 2.** Multivariable Logistic Regression of Factors Associated With Rhythm Control Strategy vs Rate Control Strategy

Variable	Adjusted OR (95% CI)	*P* value
Age	0.98 (0.98-0.98)	<.001
Male sex	1 [Reference]	
Female sex	1.08 (1.04-1.12)	<.001
Race/ethnicity		
White	1 [Reference]	
Asian	0.95 (0.84-1.08)	.43
Black	0.89 (0.83-0.94)	<.001
Latinx^a^	0.96 (0.90-1.03)	.23
Region		
West	1 [Reference]	
Midwest	0.85 (0.80-0.90)	<.001
Northeast	0.56 (0.52-0.60)	<.001
South	0.96 (0.92-1.01)	.11
Zip code–linked median household income, $		
≥100 000	1 [Reference]	
<50 000	0.83 (0.79-0.87)	<.001
50 000-99 999	0.92 (0.88-0.96)	<.001
Insurance (Medicare Advantage as reference)	1.45 (1.37-1.53)	<.001
Anticoagulation	2.54 (2.44-2.64)	<.001
Comorbidities		
HFrEF	1.67 (1.59-1.75)	<.001
HFpEF	1.41 (1.35-1.48)	<.001
Dyslipidemia	1.08 (1.03-1.14)	.003
Stroke/TIA	0.81 (0.77-0.85)	<.001
Coronary artery disease	1.05 (1.01-1.09)	.02
Chronic kidney disease	0.97 (0.92-1.01)	.11
Obesity	0.97 (0.93-1.01)	.13
Hypertension	0.95 (0.89-1.01)	.09
Diabetes	0.92 (0.88-0.96)	<.001
Peripheral vascular disease	1.06 (1.02-1.11)	.007
No. of Elixhauser comorbidities	0.98 (0.98-0.99)	<.001
No. of cardiology visits per 12 mo		
0	1 [Reference]	
1	1.62 (1.52-1.73)	<.001
>1	2.36 (2.21-2.52)	<.001

Abbreviations: HFpEF, heart failure with preserved ejection fraction; HFrEF, heart failure with reduced ejection fraction; OR, odds ratio; TIA, transient ischemic attack.

^a^ Latinx is a gender-neutral term describing persons of Latin American origin or descent.

### Catheter Ablation vs Antiarrhythmic Drugs in Rhythm Control 

Independent factors associated with catheter ablation use in multivariable analysis are summarized in Table 3. Latinx ethnicity was independently associated with less catheter ablation use (aOR, 0.73; 95% CI, 0.60-0.89; *P* = .002). Lower zip code–linked median household income was also independently associated with less catheter ablation use for those with income less than $50 000 (aOR, 0.61; 95% CI, 0.54-0.69; *P* < .001) and for those with income of $50 000 to $99 999 (aOR, 0.81; 95% CI, 0.72-0.90; *P* < .001) vs those with income of $100 000 or more. Similarly, HFrEF was independently associated with less use of catheter ablation (aOR, 0.71; 95% CI, 0.63-0.80; *P* < .001). Patients with more Elixhauser comorbidities were less likely to undergo catheter ablation (aOR, 0.89; 95% CI, 0.87-0.91; *P* < .001). Having more cardiology visits was independently associated with increased use of catheter ablation for 1 cardiology visit per 12 months (aOR, 1.84; 95% CI, 1.41-2.41; *P* < .001) and for more than 1 cardiology visit per 12 months (aOR, 3.80; 95% CI, 2.92-4.94; *P* < .001).

**Table 3.** Multivariable Logistic Regression of Factors Associated With Catheter Ablation in Rhythm Control Strategy Group

Variable	Adjusted OR (95% CI)	*P* value
Age	0.95 (0.94-0.95)	<.001
Male sex	1 [Reference]	
Female sex	0.93 (0.84-1.02)	.12
Race/ethnicity		
White	1 [Reference]	
Asian	1.18 (0.88-1.58)	.28
Black	0.86 (0.73-1.02)	.09
Latinx^a^	0.73 (0.60-0.89)	.002
Region		
West	1 [Reference]	
Midwest	0.78 (0.68-0.89)	<.001
Northeast	0.88 (0.74-1.06)	.18
South	0.89 (0.78-1.00)	.06
Zip code–linked median household income, $		
≥100 000	1 [Reference]	
<50 000	0.61 (0.54-0.69)	<.001
50 000-99 999	0.81 (0.72-0.90)	<.001
Insurance (Medicare Advantage as reference)	1.06 (0.93-1.21)	.36
Anticoagulation	3.06 (2.67-3.49)	<.001
Comorbidities		
HFrEF	0.71 (0.63-0.80)	<.001
HFpEF	0.88 (0.77-1.01)	.06
Dyslipidemia	1.28 (1.13-1.45)	<.001
Stroke/TIA	1.00 (0.86-1.16)	.99
Coronary artery disease	0.81 (0.73-0.90)	<.001
Chronic kidney disease	0.97 (0.85-1.10)	.65
Obesity	1.16 (1.05-1.29)	.005
Hypertension	0.87 (0.76-1.00)	.05
Diabetes	0.92 (0.82-1.03)	.13
Peripheral vascular disease	0.90 (0.87-1.01)	.08
No. of Elixhauser comorbidities	0.89 (0.87-0.91)	<.001
No. of cardiology visits per 12 mo		
0	1 [Reference]	
1	1.84 (1.41-2.41)	<.001
>1	3.80 (2.92-4.94)	<.001

Abbreviations: HFpEF, heart failure with preserved ejection fraction; HFrEF, heart failure with reduced ejection fraction; OR, odds ratio; TIA, transient ischemic attack.

^a^ Latinx is a gender-neutral term describing persons of Latin American origin or descent.

Among the 3500 patients who underwent catheter ablation, 1651 (47.2%) had no AAD prescription filled prior to catheter ablation, 1513 (43.2%) had 1 prescription filled, 292 (8.3%) had two, and 44 (1.3%) had 3 or more different AAD prescriptions filled. No difference in the number of unique AAD prescriptions filled from the time of paroxysmal AF diagnosis to catheter ablation was observed between the different income groups or between racial/ethnic groups. However, female patients filled more unique AAD prescriptions between the time of paroxysmal AF diagnosis and catheter ablation vs male patients (618 [44.3%] vs 1033 [49.1%] with no AAD prescription filled; 615 [44.1%] vs 898 [42.7%] with 1; 141 [10.1%] vs 151 [7.2%] with 2; 21 [1.5%] vs 23 [1.0%] with ≥3 AAD prescriptions filled prior to catheter ablation).

## Discussion

To our knowledge, this study is the first to investigate for the presence of disparities in paroxysmal AF management and catheter ablation by race/ethnicity and socioeconomic status among patients with commercial insurance in the US. Between 2016 and 2019, we found that the rate of AAD and catheter ablation use increased among patients with paroxysmal AF, yet the rate of catheter ablation remained low (only 3.2% of patients with incident paroxysmal AF), particularly among patients with HFrEF. Among a commercially insured population, significant disparities in treatment strategy based on race/ethnicity and socioeconomic status were observed. Black race and lower zip code–linked median household income were independently associated with lower use of a rhythm control strategy. Latinx ethnicity and lower zip code–linked median household income were independently associated with lower use of catheter ablation, even among patients in whom a rhythm control strategy was pursued.

In this study, Black race was independently associated with lower use of a rhythm control strategy. However, among those in whom a rhythm control strategy was pursued, Black race was not associated with lower rates of catheter ablation. Reduced access to specialty care, including cardiovascular care, has been demonstrated among Black patients.^19,20,21^ Although we adjusted for the number of outpatient cardiology visits per 12 months, these treatment differences may reflect barriers in accessing specialty cardiology care, particularly referral to a cardiac electrophysiologist for the management of paroxysmal AF, among Black patients. Black patients may receive more paroxysmal AF care from general medicine and cardiology practitioners, who may be comfortable prescribing commonly used rate control agents but may be less comfortable prescribing AADs because of their adverse and toxic effects. However, for Black patients who are able to access specialized care in which clinicians are comfortable implementing rhythm control strategies, there did not appear to be differences in pursuing catheter ablation. The number of cardiology visits was strongly associated with rhythm control and catheter ablation use. Addressing structural racism and the barriers to accessing cardiology care for marginalized patient groups is needed.^19,20,21^

Latinx patients were significantly less likely to undergo catheter ablation for treatment of paroxysmal AF, even among patients for whom a rhythm control strategy was selected. These findings are consistent with those of a previous study of Medicare patients^16^ that showed less catheter ablation use among Latinx patients with AF. Decreased rates of other specialized cardiovascular procedures, such as coronary catheterization and device implantation, among Latinx patients are well documented.^14,15,22^ We were unable to account for language in the database, but appreciable inequities in health care use, particularly for specialized procedures and care, among non–English-speaking Latinx patients have been reported previously^15^ and may have played a role in the findings of the present study.

The differences we observed in the care of Black and Latinx patients with paroxysmal AF were in the setting of a population with 100% commercial insurance and were adjusted for outpatient cardiology visits, which raise concerns about bias in care delivery.^23^ Patient advocacy may be a factor in treatment decisions given that White patients may more frequently advocate for rhythm control strategies and potentially curative ablation procedures. Racial and ethnic differences in self-advocacy have been observed and are likely reflective of a long history of discrimination against racial/ethnic minority groups in the US health care system.^24^ Clinician interactions with patients from racial/ethnic minority groups have been characterized by less patient input regarding treatment decisions.^25^ Lower rates of patient activation (ie, confidence to engage in health care and treatment decisions among marginalized patients) because of frequent and recurrent inequitable treatment by the health system may play a role in the demonstrated differences in paroxysmal AF management.^26,27^

We also found significant differences in the use of catheter ablation by socioeconomic status. Overall, patients with a zip code–linked median household income of less than $50 000 were 17% less likely to receive a rhythm control strategy and were 39% less likely to receive catheter ablation compared with those with a median household income of $100 000 or more. In addition to barriers to accessing more advanced cardiac electrophysiology care, affordability of AADs and catheter ablation likely contributed to our findings. Similar to findings in patients from racial/ethnic minority groups, it has been shown that novel therapies are less likely to be used among patients with lower income.^28^ This finding may also reflect clinician bias; clinicians may preferentially offer catheter ablation to patients with higher income because of perceptions that patients with lower income are unable to afford such treatment.^29^

We found that the rate of catheter ablation was low (3.2%) for the overall population and for patients with HFrEF (2.8%), which was surprising. Given the increasing evidence of benefits in this population, including improved mortality, decreased rate of hospitalization for heart failure, and increased left ventricular ejection fraction,^5,6,7,8^ the American College of Cardiology/American Heart Association updated AF guidelines in 2019 to include a recommendation that catheter ablation may be reasonable to use for this population.^4^ The study period preceded some of the key trials that led to this change in guidelines.^6,7,8^ However, given more recent evidence of improved cardiovascular outcomes associated with pursuit of an early rhythm control strategy among all patients with AF regardless of symptoms,^5^ the implementation of strategies that address inequitable access to rhythm control strategies is important moving forward.

### Limitations

This study has several limitations. Because we obtained data from an administrative database, we were unable to fully characterize the complexity of patient care decisions. We were unable to describe how patient preference, clinician expertise, nuanced clinical decision-making for individual patients, and practice patterns impacted treatment decisions. The differences in patient preferences for invasive procedures may have been a factor in the differences in catheter ablation use. Decreased preference for invasive cardiac procedures among non-White patients has been shown to be mediated by lack of familiarity with these procedures, which in large part is related to differences and biases in clinician communication.^30^ Improved communication regarding catheter ablation and its benefits for marginalized patients may improve equitable uptake.

The presence of symptoms or AF burden has been a key factor for many clinicians to pursue rhythm control and catheter ablation in paroxysmal AF. We were unable to discern AF burden or if patients had symptomatic AF with this data set. However, we suspect that these findings reflect inequities in care because we do not expect Black or Latinx patients or patients with lower income to experience less symptomatic disease. Some evidence suggests that non-White patients may have more symptoms with a greater impact on quality of life.^31^ In addition, certain patients, such as those from a lower socioeconomic status, likely have a higher threshold to present for care unless substantial symptoms are present. Furthermore, even among those for whom a rhythm control strategy was selected (and therefore were presumably symptomatic or had other important indications for rhythm control), catheter ablation was used less frequently for patients from racial/ethnic minority groups and patients with a lower income. Alternatively, the differences that we observed may reflect the overuse of therapies, such as catheter ablation, when not clinically indicated for White patients with higher income, which we could not discern from the database.

Patients with paroxysmal AF were selected using *ICD-10* codes only given that *ICD-10* requires specifying AF type (eg, paroxysmal, persistent), whereas *ICD-9* does not. Nevertheless, it is still possible that some patients were misclassified. The findings may not apply to populations with persistent and long-standing persistent AF.

The population studied had commercial insurance, with a large proportion covered by Medicare Advantage, and was slightly older than the general population with AF. These characteristics may limit generalizability for other payer groups or younger populations. However, we anticipate that the observed differences may be even greater among those with Medicaid or without health care insurance.

Despite these limitations, the results of this study highlight important treatment differences based on race/ethnicity and socioeconomic status. These results emphasize the need for continued efforts to ensure that access to all AF treatments is equitable.

## Conclusions

In this cohort study of a diverse, commercially insured population, rates of AAD and catheter ablation use for the management of patients with paroxysmal AF increased between 2016 and 2019. However, catheter ablation use remained low, particularly among patients with HFrEF. Black race and lower zip code–linked median household income were independently associated with less use of a rhythm control strategy, whereas Latinx ethnicity and lower income were independently associated with lower use of catheter ablation. These findings suggest that both racial/ethnic and socioeconomic inequities may be present in accessing advanced AF treatments. Additional studies to better understand the barriers to these AF therapies to ensure equitable access to all therapies are warranted.

## Notes

Supplement. **eFigure.** Cumulative Rates of Treatment With AADs and Catheter Ablation for Paroxysmal Atrial Fibrillation Among Patients With HFrEF (A) and Cumulative Rates of Treatment With Catheter Ablation for Paroxysmal Atrial Fibrillation by Race/Ethnicity Among Patients With HFrEF (B)

## References

[1] Wolf PA, Benjamin EJ, Belanger AJ, Kannel WB, Levy D, D’Agostino RB. Secular trends in the prevalence of atrial fibrillation: the Framingham Study. Am Heart J. 1996;131(4):790-795. doi:10.1016/S0002-8703(96)90288-4 8721656

[2] January CT, Wann LS, Alpert JS, ; ACC/AHA Task Force Members. 2014 AHA/ACC/HRS guideline for the management of patients with atrial fibrillation: executive summary: a report of the American College of Cardiology/American Heart Association Task Force on practice guidelines and the Heart Rhythm Society. Circulation. 2014;130(23):2071-2104. doi:10.1161/CIR.0000000000000040 24682348

[3] Mark DB, Anstrom KJ, Sheng S, ; CABANA Investigators. Effect of catheter ablation vs medical therapy on quality of life among patients with atrial fibrillation: the CABANA randomized clinical trial [published correction appears in *JAMA*. 2019;321(23):2370]. JAMA. 2019;321(13):1275-1285. doi:10.1001/jama.2019.0692 30874716 PMC6450275

[4] January CT, Wann LS, Calkins H, . 2019 AHA/ACC/HRS focused update of the 2014 AHA/ACC/HRS Guideline for the Management of Patients With Atrial Fibrillation: a report of the American College of Cardiology/American Heart Association Task Force on Clinical Practice Guidelines and the Heart Rhythm Society. J Am Coll Cardiol. 2019;74(1):104-132. doi:10.1016/j.jacc.2019.01.011 30703431

[5] Kirchhof P, Camm AJ, Goette A, ; EAST-AFNET 4 Trial Investigators. Early rhythm-control therapy in patients with atrial fibrillation. N Engl J Med. 2020;383(14):1305-1316. doi:10.1056/NEJMoa2019422 32865375

[6] Calkins H, Kuck KH, Cappato R, ; Heart Rhythm Society Task Force on Catheter and Surgical Ablation of Atrial Fibrillation. 2012 HRS/ EHRA/ECAS expert consensus statement on catheter and surgical ablation of atrial fibrillation: recommendations for patient selection, procedural techniques, patient management and follow-up, definitions, endpoints, and research trial design: a report of the Heart Rhythm Society (HRS) Task Force on Catheter and Surgical Ablation of Atrial Fibrillation. Developed in partnership with the European Heart Rhythm Association (EHRA), a registered branch of the European Society of Cardiology (ESC) and the European Cardiac Arrhythmia Society (ECAS); and in collaboration with the American College of Cardiology (ACC), American Heart Association (AHA), the Asia Pacific Heart Rhythm Society (APHRS), and the Society of Thoracic Surgeons (STS). Heart Rhythm. 2012;9(4):632-696. doi:10.1016/j.hrthm.2011.12.016 22386883

[7] Marrouche NF, Brachmann J, Andresen D, ; CASTLE-AF Investigators. Catheter ablation for atrial fibrillation with heart failure. N Engl J Med. 2018;378(5):417-427. doi:10.1056/NEJMoa1707855 29385358

[8] Di Biase L, Mohanty P, Mohanty S, . Ablation versus amiodarone for treatment of persistent atrial fibrillation in patients with congestive heart failure and an implanted device: results from the AATAC multicenter randomized trial. Circulation. 2016;133(17):1637-1644. doi:10.1161/CIRCULATIONAHA.115.019406 27029350

[9] Prabhu S, Taylor AJ, Costello BT, . Catheter ablation versus medical rate control in atrial fibrillation and systolic dysfunction: the CAMERA-MRI Study. J Am Coll Cardiol. 2017;70(16):1949-1961. doi:10.1016/j.jacc.2017.08.041 28855115

[10] Al Halabi S, Qintar M, Hussein A, . Catheter ablation for atrial fibrillation in heart failure patients: a meta-analysis of randomized controlled trials. JACC Clin Electrophysiol. 2015;1(3):200-209. doi:10.1016/j.jacep.2015.02.018 26258174 PMC4525704

[11] Smedley BD; Institute of Medicine. Unequal Treatment: Confronting Racial and Ethnic Disparities in Health Care. The National Academies Press; 2003. 25032386

[12] Essien UR, Holmes DN, Jackson LR II, . Association of race/ethnicity with oral anticoagulant use in patients with atrial fibrillation: findings from the Outcomes Registry for Better Informed Treatment of Atrial Fibrillation II. JAMA Cardiol. 2018;3(12):1174-1182. doi:10.1001/jamacardio.2018.3945 30484833 PMC6583087

[13] Nathan AS, Geng Z, Dayoub EJ, . Racial, ethnic, and socioeconomic inequities in the prescription of direct oral anticoagulants in patients with venous thromboembolism in the United States. Circ Cardiovasc Qual Outcomes. 2019;12(4):e005600. doi:10.1161/CIRCOUTCOMES.119.005600 30950652 PMC9119738

[14] Carlisle DM, Leake BD, Shapiro MF. Racial and ethnic differences in the use of invasive cardiac procedures among cardiac patients in Los Angeles County, 1986 through 1988. Am J Public Health. 1995;85(3):352-356. doi:10.2105/AJPH.85.3.352 7892918 PMC1614877

[15] Hess PL, Hernandez AF, Bhatt DL, . Sex and race/ethnicity differences in implantable cardioverter-defibrillator counseling and use among patients hospitalized with heart failure: findings from the Get With the Guidelines-Heart Failure Program. Circulation. 2016;134(7):517-526. doi:10.1161/CIRCULATIONAHA.115.021048 27492903

[16] Hoyt H, Nazarian S, Alhumaid F, . Demographic profile of patients undergoing catheter ablation of atrial fibrillation. J Cardiovasc Electrophysiol. 2011;22(9):994-998. doi:10.1111/j.1540-8167.2011.02043.x 21385269

[17] Bhave PD, Lu X, Girotra S, Kamel H, Vaughan Sarrazin MS. Race- and sex-related differences in care for patients newly diagnosed with atrial fibrillation. Heart Rhythm. 2015;12(7):1406-1412. doi:10.1016/j.hrthm.2015.03.031 25814418 PMC4787261

[18] Elixhauser A, Steiner C, Harris DR, Coffey RM. Comorbidity measures for use with administrative data. Med Care. 1998;36(1):8-27. doi:10.1097/00005650-199801000-00004 9431328

[19] Cook NL, Ayanian JZ, Orav EJ, Hicks LS. Differences in specialist consultations for cardiovascular disease by race, ethnicity, gender, insurance status, and site of primary care. Circulation. 2009;119(18):2463-2470. doi:10.1161/CIRCULATIONAHA.108.825133 19398667

[20] Eberly LA, Richterman A, Beckett AG, . Identification of racial inequities in access to specialized inpatient heart failure care at an Academic Medical Center. Circ Heart Fail. 2019;12(11):e006214. doi:10.1161/CIRCHEARTFAILURE.119.006214 31658831 PMC7183732

[21] Breathett K, Liu WG, Allen LA, . African Americans are less likely to receive care by a cardiologist during an intensive care unit admission for heart failure. JACC Heart Fail. 2018;6(5):413-420. doi:10.1016/j.jchf.2018.02.015 29724363 PMC5940011

[22] Fiscella K, Franks P, Doescher MP, Saver BG. Disparities in health care by race, ethnicity, and language among the insured: findings from a national sample. Med Care. 2002;40(1):52-59. doi:10.1097/00005650-200201000-00007 11748426

[23] Dehon E, Weiss N, Jones J, Faulconer W, Hinton E, Sterling S. A systematic review of the impact of physician implicit racial bias on clinical decision making. Acad Emerg Med. 2017;24(8):895-904. doi:10.1111/acem.13214 28472533

[24] Wiltshire J, Cronin K, Sarto GE, Brown R. Self-advocacy during the medical encounter: use of health information and racial/ethnic differences. Med Care. 2006;44(2):100-109. doi:10.1097/01.mlr.0000196975.52557.b7 16434908

[25] Johnson RL, Roter D, Powe NR, Cooper LA. Patient race/ethnicity and quality of patient-physician communication during medical visits. Am J Public Health. 2004;94(12):2084-2090. doi:10.2105/AJPH.94.12.2084 15569958 PMC1448596

[26] Eneanya ND, Olaniran K, Xu D, . Health literacy mediates racial disparities in cardiopulmonary resuscitation knowledge among chronic kidney disease patients. J Health Care Poor Underserved. 2018;29(3):1069-1082. doi:10.1353/hpu.2018.0080 30122684 PMC6249689

[27] Eneanya ND, Winter M, Cabral H, . Health literacy and education as mediators of racial disparities in patient activation within an elderly patient cohort. J Health Care Poor Underserved. 2016;27(3):1427-1440. doi:10.1353/hpu.2016.0133 27524777 PMC5718153

[28] Sholzberg M, Gomes T, Juurlink DN, Yao Z, Mamdani MM, Laupacis A. The influence of socioeconomic status on selection of anticoagulation for atrial fibrillation. PLoS One. 2016;11(2):e0149142. doi:10.1371/journal.pone.0149142 26914450 PMC4767939

[29] van Ryn M, Burke J. The effect of patient race and socio-economic status on physicians’ perceptions of patients. Soc Sci Med. 2000;50(6):813-828. doi:10.1016/S0277-9536(99)00338-X 10695979

[30] Whittle J, Conigliaro J, Good CB, Joswiak M. Do patient preferences contribute to racial differences in cardiovascular procedure use? J Gen Intern Med. 1997;12(5):267-273. doi:10.1007/s11606-006-5062-0 9159695 PMC1497106

[31] Gleason KT, Nazarian S, Dennison Himmelfarb CR. Atrial fibrillation symptoms and sex, race, and psychological distress: a literature review. J Cardiovasc Nurs. 2018;33(2):137-143. doi:10.1097/JCN.0000000000000421 28628500 PMC5733721

