# Supplementary material for: Racial/Ethnic and Socioeconomic Disparities in Management of Incident Paroxysmal Atrial Fibrillation
Source: JAMA Netw Open. 2021 Feb 26;4(2):e210247. doi: 10.1001/jamanetworkopen.2021.0247 (PMC7910819; doi:10.1001/jamanetworkopen.2021.0247)
Supplement: Supplement. — eFigure. Cumulative Rates of Treatment With AADs and Catheter Ablation for Paroxysmal Atrial Fibrillation Among Patients With HFrEF (A) and Cumulative Rates of Treatment With Catheter Ablation for Paroxysmal Atrial Fibrillation by Race/Ethnicity Among Patients With HFrEF (B) [file jamanetwopen-e210247-s001.pdf]

Supplementary Online Content

Eberly LA, Garg L, Yang L, et al. Racial/ethnic and socioeconomic disparities in management of incident paroxysmal atrial fibrillation. JAMA Netw Open. 2021;4(2):e210247. doi:10.1001/jamanetworkopen.2021.0247

eFigure. Cumulative Rates of Treatment with AADs and Catheter Ablation for Paroxysmal Atrial Fibrillation Among Patients with HFrEF (A) and Cumulative Rates of Treatment with Catheter Ablation for Paroxysmal Atrial Fibrillation by Race/Ethnicity Among Patients with HFrEF (B)

This supplementary material has been provided by the authors to give readers additional information about their work.

©2021 Eberly LA et al. *JAMA Network Open.*

eFigure. Cumulative Rates of Treatment with AADs and Catheter Ablation for Paroxysmal Atrial Fibrillation Among Patients with HFrEF (A) and Cumulative Rates of Treatment with Catheter Ablation for Paroxysmal Atrial Fibrillation by Race/Ethnicity Among Patients with HFrEF (B)

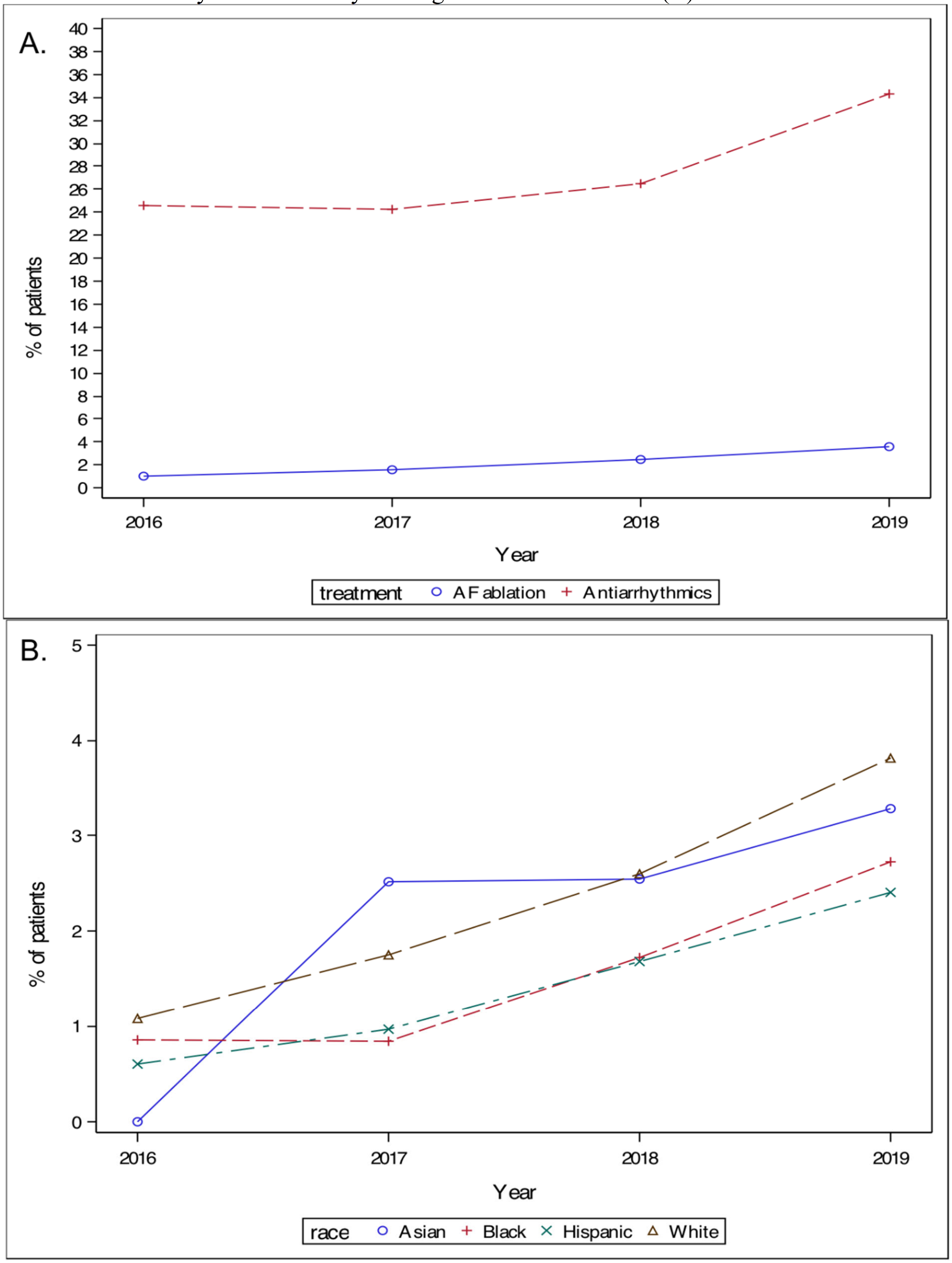

©2021 Eberly LA et al. *JAMA Network Open.*
